# Ultrasound during Advanced Life Support—Help or Harm?

**DOI:** 10.3390/diagnostics14060593

**Published:** 2024-03-11

**Authors:** Adrian Goudie, Michael Blaivas, Rudolf Horn, Wan-Ching Lien, Guido Michels, Daniel Wastl, Christoph Frank Dietrich

**Affiliations:** 1 Department of Emergency Medicine, Fiona Stanley Hospital, Murdoch 6150, Australia; adrian.goudie@health.wa.gov.au; 2 Department of Medicine, University of South Carolina School of Medicine, Columbia, SC 29209, USA; mike@blaivas.org; 3 Center da sandà Val Müstair, Santa Maria, 7537 Val Müstair, Switzerland; rudolf@horn-ch.org; 4 Department of Emergency Medicine, College of Medicine, National Taiwan University, Taipei 10617, Taiwan; dtemer17@yahoo.com.tw; 5 Department of Emergency Medicine, National Taiwan University Hospital, Taipei 10617, Taiwan; 6 Notfallzentrum, Krankenhaus der Barmherzigen Brüder Trier, 54292 Trier, Germany; guidomichels@gmx.de; 7 Krankenhaus Nordwest, 60488 Frankfurt, Germany; daniel-wastl@t-online.de; 8 Department Allgemeine Innere Medizin (DAIM), Kliniken Hirslanden Beau Site, Salem und Permanence, 3013 Bern, Switzerland

**Keywords:** ultrasound, cardiopulmonary resuscitation, cardiac arrest, echocardiography

## Abstract

Ultrasound is used in cardiopulmonary resuscitation (CPR) and advanced life support (ALS). However, there is divergence between the recommendations of many emergency and critical care societies who support its use and the recommendations of many international resuscitation organizations who either recommend against its use or recommend it only in limited circumstances. Ultrasound offers potential benefits of detecting reversable causes of cardiac arrest, allowing specific interventions. However, it also risks interfering with ALS protocols and increasing unhelpful interventions. As with many interventions in ALS, the evidence base for ultrasound use is weak, and well-designed randomized trials are needed. This paper reviews the current theory and evidence for harms and benefits.

## 1. Introduction

Echocardiography in cardiopulmonary resuscitation (CPR) has been described for over four decades [1]. With advances in technology resulting in small and portable ultrasound machines, the use of ultrasound in unstable and critically ill patients (often referred to as “point-of-care ultrasound” (PoCUS) and “basic” or “focused” echocardiography) has been advocated by multiple societies and groups and is now considered an essential component of training for emergency and critical care physicians [2,3,4,5,6]. Advocates describe the use of ultrasound during advanced life support (ALS) in the pre-arrest, arrest and post-arrest settings to assist diagnosis, guide management, and provide prognostic information. They recommend, either implicitly or explicitly, its use during CPR. However, many guidelines by major national and international resuscitation societies are either equivocal or warn against the general use of ultrasound in ALS [7,8,9,10]. These guidelines cite lack of evidence of effectiveness and potential harm from performing ultrasound during ALS resuscitation. 

A narrative review was therefore performed to assist understanding of these divergent recommendations, focusing on the use of ultrasound during CPR.

## 2. Recommendations by Resuscitation Societies

The International Liaison Committee on Resuscitation (ILCOR) provides a forum for liaison between principal resuscitation organizations worldwide. In its 2020 Consensus statement, it warned against the use of PoCUS for prognostication in CPR [11]. In 2022, it warned against routine use of PoCUS during CPR to diagnose reversible causes, although “if PoCUS can be performed by experienced personnel without interrupting CPR it may be considered as an additional diagnostic tool when clinical suspicion for a specific reversible cause is present” [8].

The 2021 European Resuscitation Council (ERC) Guidelines on ALS and the 2020 American Heart Association (AHA) Guidelines emphasize the requirement for a skilled operator and the need to minimize interruptions during chest compressions [7,9]. Australian and New Zealand guidelines state ultrasound may be considered if it does not interfere with standard ALS [10]. ERC recommends the use of echocardiography in the setting of a catheterization laboratory, operating room, traumatic cardiac arrest, pregnancy, and after return of spontaneous circulation [12,13]. ILCOR and AHA both recommend against the use of ultrasound for prognostication [9,11] whilst the ERC states PoCUS should not be used as a sole indicator for terminating CPR [7]. 

Despite these recommendations, all the resuscitation organizations recognize that there is increasing use of ultrasound in resuscitation [7,9,11]. This increasing use is supported by many emergency and critical care societies, although most do not specifically provide recommendations regarding use during CPR. The International Federation for Emergency Medicine Consensus Statement on sonography in hypotension and cardiac arrest recommends the use of ultrasound, noting that cardiac views must be performed during the rhythm check without causing prolonged interruption to chest compressions [14]. Korean resuscitation guidelines recommend using PoCUS “if available and not interfering with CPR” [15]. Many emergency medicine societies in America, Australasia, United Kingdom, and Asia require training in ultrasound including its use during cardiac arrest, although they do not have specific statements recommending its use in ALS [4,6,16,17]. Some groups advocate for transesophageal ultrasound, claiming that it can avoid or overcome the limitations of transthoracic echocardiogram and provide further information [18].

## 3. Use of Ultrasound for Diagnosis in Resuscitation

In cardiopulmonary resuscitation, the following reversible causes of cardiac arrest must be assessed [Table 1] [19].

Ultrasound findings suggestive of hypovolemia are a small inferior vena cava which “collapses” significantly during expiration phase of positive pressure ventilation (or during inspiration if the patient is spontaneously breathing), a small left ventricle which may be hyperdynamic (if contracting) and a small right ventricle. In addition, hypovolemia may be supported by identifying large amounts of free fluid in the appropriate clinical context (using the eFAST or RUSH protocol) [20]. 

Cardiac tamponade is identified by the presence of a pericardial effusion. Pre-arrest, collapse of the right atrium and right ventricle are seen, associated with an enlarged IVC (>2 cm) that collapses little throughout the respiratory cycle [20]. In the setting of resuscitation, the presence of a medium-to-large pericardial effusion should be assumed to be causing tamponade, whilst remembering that a smaller, loculated effusion can also cause tamponade. It is important to note that a very large pericardial effusion in chronic pericarditis is often tolerated well, in contrast to a small but acutely accumulating effusion, which results in a tamponade. Ultrasound should be used to guide pericardiocentesis. A subcostal, apical, or parasternal approach can be used, depending on the ultrasound findings [21,22]. Using echocardiography, success rates are high and complication rates are very low, with major complication rates of 1–3% (although these series are not restricted to pericardiocentesis performed in the resuscitation setting) [23,24,25,26,27].

Myocardial infarction may be suggested by regional wall motion abnormality in a contracting heart [28,29]. However, it should be cautioned that myocardial dysfunction can be seen in one third of post-cardiac-arrest patients without myocardial infarction or previous cardiac disease post resuscitation, including global dysfunction, regional wall motion abnormality, and the Takotsubo pattern [30]. In a series of 237 patients post return of spontaneous circulation (ROSC), 17 were described as having left ventricular regional wall motion abnormalities, 9 of whom were subsequently treated with percutaneous coronary intervention [31]. In this situation, a myocardial infarction can only be suspected with ultrasound and certainly not ruled out.

Right ventricular enlargement, pressure overload and dysfunction, and hemodynamic instability may suggest pulmonary embolism as a cause for cardiac arrest. Findings of an increased right ventricle/left ventricle ratio, flattening of the intraventricular septum (resulting in a “D” shape on short axis views), elevated pulmonary arterial pressure, and McConnell’s sign (preserved function of the right ventricular apex with reduced function of the mid right ventricular wall) are all consistent with massive pulmonary embolism [32]. However, right ventricular enlargement can occur due to cardiac arrest itself, and recent ILCOR and ERC guidelines state that it should not be used as a sole criterion for diagnosing massive pulmonary embolism [7,11,33,34]. Further, pre-existing pulmonary or cardiac disease can cause right ventricular dilation, pulmonary hypertension, and right ventricular dysfunction [32]. In a post-ROSC cohort, 21/237 patients underwent computerized tomography based on right ventricular echo findings, 11 of whom were diagnosed with pulmonary embolism [31]. Another series of post-ROSC patients reported that 59% had right ventricular dysfunction on initial post-arrest echocardiograms [35]. Therefore, treating a cardiac arrest patient with thrombolytics for presumed massive pulmonary embolism may be reserved for cases with other compelling evidence such as identification of thrombus in the right heart or pulmonary arteries during resuscitation. Direct evidence of pulmonary or venous thrombus may be seen on ultrasound in the pulmonary trunk, right ventricle, or atrium or the deep veins (most commonly the proximal leg veins). In these circumstances, current guidelines on pulmonary embolism suggest that if pulmonary embolism is suspected and a deep vein thrombosis (DVT) is seen on venous ultrasound, a diagnosis of pulmonary embolism can be accepted [36].

Ultrasound to detect or exclude pneumothorax is well established with a high sensitivity and specificity [37], although most studies are not performed in the unstable or arresting patient. Ultrasound signs suggestive (but not diagnostic) of a pneumothorax include lack of lung sliding or lung pulse and absence of comet tail artefacts [38]. If the pneumothorax is not too large, usually a lung point can be seen at the edge of the pneumothorax (the most specific ultrasound sign of a pneumothorax); however, in a tension pneumothorax, this point may be too posterior to be examined, particularly in the setting of resuscitation. Other reported findings that may be noted in a tension pneumothorax are mediastinal shift and IVC dilation [39].

Ultrasound is the primary diagnostic tool used to distinguish between pulseless electrical activity (PEA) and pseudo-PEA. In pseudo-PEA, no pulse can be felt, but there is organized cardiac activity. As palpation of pulses is known to be unreliable during resuscitation, ultrasound can be used to examine the arterial system to detect pulses to also distinguish between PEA and pseudo-PEA, either by Doppler or B-mode imaging [40,41,42].

Pseudo-PEA can be due to profound depressed myocardial contractility but also due to tachydysrhythmias, hypovolemic shock, pulmonary embolism, tension pneumothorax, or cardiac tamponade [43]. These different causes have different treatments, and in addition to potentially delaying effective treatments, some animal studies suggest cardiac compression may be ineffective or even harmful by reducing cardiac output [13,44,45]. For pseudo-PEA patients without other reversible causes, some studies have suggested different treatment algorithms [46,47]. The use of ultrasound increases the rate of diagnosis of ‘reversible causes’ [48], although they remain the minority of arrest cases [49,50,51].

## 4. Use of Ultrasound for Prognosis during ALS

Many case series have described using ultrasound for prognosis during resuscitation, based on assessment of cardiac contraction. A study examined the reliability of readers’ interpretation of this finding, with only poor interrater agreement [52]. Whilst all series report a worse outcome for patients with absent contractility (with many earlier series reporting no survival), all are described as having poor interrater reliability and a moderate-to-high risk of bias [53,54]. All series are unblinded and thus risk the decision to cease resuscitation being influenced by the finding (confirmation bias resulting in a “self-fulfilling prophecy”). More recent series demonstrate a very low (but not zero) chance of survival if there is absent cardiac motion, with a lower chance of survival compared to patients with cardiac motion [53,54,55,56]. One study showed a weak correlation between the degree of contractility and outcome [57]. The presence of intracardiac clot has also been described as predicting a very poor outcome [58]. A metanalysis of traumatic cardiac arrest had similar findings, with zero survival to hospital discharge in patients without cardiac activity; however, these studies were again unblinded [59].

The ILCOR guidelines, based on its systematic review, therefore recommended against the use of PoCUS for prognostication [11]. The ERC guidelines state the following: “Do not use PoCUS for assessing contractility of the myocardium as the sole indicator for terminating CPR” [7].

## 5. Other Uses of Ultrasound in CPR

There is uncertainty as to the optimal hand positioning for chest compression during CPR. Studies have demonstrated that positioning using the traditionally recommended position (“middle of the victim’s chest/lower half of the sternum”) is often situated over the left ventricular outflow tract and aortic root and that positioning over the lower third of the sternum may be improve some physiological parameters [60]. Ultrasound (especially transesophageal echocardiography) can guide hand positioning to ensure that maximal compression occurs over the left ventricle, which has been shown to improve parameters and outcomes in animal models [61], although no outcome studies have been performed. Ultrasound has been described being used to confirm endotracheal intubation during resuscitation, with confirmation being faster than by capnography [28].

## 6. Potential Harm of Ultrasound during ALS

Potential harm can occur from any intervention. Errors in diagnosis (false positive and false negative results) may result in inappropriate interventions being performed or potentially helpful interventions not occurring. Studies have shown rapid learning curves for ultrasound use in ALS, including during a simulated scenario after a German ALS course which did not include any specific teaching on ultrasound [62]. Studies have also demonstrated that non-medical staff can also rapidly learn the use of ultrasound in ALS [63].

The major concern for use of ultrasound during cardiac arrest is that it may increase the length of pauses in chest compression. Studies analyzing actual arrests have demonstrated prolongation of pulse check time, often beyond the recommended limit of 10 s [64,65]. In response to this, many protocols have been described and shown to reduce this delay [66,67,68,69]. Shortening the time for ultrasound may, however, potentially lower the diagnostic accuracy, although no studies have been performed to test this. The use of transesophageal echocardiography can also reduce the duration of pauses [70].

## 7. Outcome Trials of Ultrasound in ALS

Despite theoretical benefits and risks, ultimately, any intervention is judged based on demonstrating benefit or harm. For ultrasound use in ALS, there are only two studies that report outcomes, neither of which were randomized. The results of these studies are presented in Table 2.

Lien et al., describes a convenience sample of 236 patients, of whom 190 had ultrasound if a qualified clinician was present. The ultrasound group had a lower rate of ROSC, and there was no significant difference between survival to hospital discharge and favorable neurological outcome [68]. Pyo et al. retrospectively studied groups before and after introducing an ultrasound protocol. The use of ultrasound increased from 32% to 79%, and examined more structures. The study demonstrated no difference in outcomes, but an increase in resuscitative procedures (from 0.6% to 4.9%) [48].

Recommendations for the timing of the use of ultrasound in resuscitation are variable. Non-randomized studies are at risk of selection bias as ultrasound may be used more often in those with expected poorer outcomes. Ultrasound may not be used in cases with shorter resuscitation, either due to the protocol (e.g., “use ultrasound if no ROSC by xx minutes” [15,68]) or the time taken for the operator and equipment to be available, thereby excluding patients who have early ROSC from the ultrasound cohort. Ultrasound may be used less frequently for patients with shockable rhythms. If the comparison (non-ultrasound) group includes these patients, it may bias the results in favor of not using ultrasound. Ultrasound use may also be a marker for other variations in resuscitation practice, confounding results in non-randomized trials.

## 8. Discussion

Although the most important outcomes of resuscitation trials are survival and survival with good neurological outcome, other potential benefits and harms can be considered. These include resource utilization, which ultrasound may increase (for example, due to increased unhelpful procedures or prolonging futile resuscitation efforts) or may decrease (for example, improving prognostication and therefore reducing unsuccessful resuscitation times). In addition to the early detection of reversible causes, ultrasound may be able to improve CPR quality (although this would be a surrogate measure rather than a patient-orientated outcome). No randomized studies currently exist to answer these questions as to whether there is an overall benefit or harm to patient-orientated or important system-level outcomes. When considering the level of evidence available for ultrasound during ALS, it should be noted that many other recommendations regarding ALS are based upon poor-quality evidence. This emphasizes the need for high-quality trials to determine what, if any, role ultrasound should play in ALS protocols. Such a trial would optimally be randomized (either pre-hospital or at hospital presentation for out-of-hospital cardiac arrest, or as soon as identified for in-hospital cardiac arrest patients) and the results analyzed on an intention-to-treat basis.

## 9. Conclusions

Currently, like many interventions performed during ALS, ultrasound use is primarily based on potential and theoretical benefit. Concerns about potential harms, particularly prolonged pauses in chest compression, must be carefully considered and avoided by strict adherence to time limits on pauses or possibly the use of TEE. Future studies are required to determine if ultrasound should or should not become a standard component in ALS protocols.

## Figures and Tables

**Table 1:** Reversible causes of cardiac arrest (5-H/5-T) according to the AHA-Algorithm. For those marked with an asterisk (*), ultrasound can be used for diagnosis.

Reversible Causes of Cardiac Arrest (5-H/5-T), According to the AHA-Algorithm	
Hypovolemia *	Tension pneumothorax *
Hypoxia	Tamponade (cardiac) *
Hypothermia	Thrombosis (myocardial infarction) *
H+ ions (acidosis)	Thrombosis (pulmonary) *
Hypo-/hyperkalemia	Toxins

**Table 2:** Outcome of ultrasound in ALS.

	Number (Ultrasound/No Ultrasound)	ROSC	Survival to Hospital Discharge	Favorable Neurological Outcome
Lien et al. [68]	236 (190/46)	36% v 52% *p* = 0.04	11% v 15% *p* = 0.37	5% v 9% *p* = 0.23
Pyo et al. [48]	Pre protocol: 149 (101/48) Post protocol: 185 (39/146)		12% v 12% *p* = 0.81	6% v 8% *p* = 0.51
